# A pilot study comparing two weight loss maintenance interventions among low-income, mid-life women

**DOI:** 10.1186/1471-2458-13-653

**Published:** 2013-07-15

**Authors:** Carmen D Samuel-Hodge, Larry F Johnston, Ziya Gizlice, Beverly A Garcia, Sara C Lindsley, Alison D Gold, Danielle F Braxton, Thomas C Keyserling

**Affiliations:** 1 Department of Nutrition, Gillings School of Global Public Health and School of Medicine, University of North Carolina at Chapel Hill, Chapel Hill, NC, USA; 2 Department of Medicine, School of Medicine, University of North Carolina at Chapel Hill, Chapel Hill, NC, USA; 3 Center for Health Promotion and Disease Prevention, University of North Carolina at Chapel Hill, 1700 Martin Luther King Jr. Blvd, Chapel Hill, NC, USA; 4 Hudson River Healthcare, Inc, Peekskill, NY, USA

**Keywords:** Obesity, Low-income women, Weight loss maintenance, Intervention

## Abstract

**Background:**

Despite high obesity prevalence rates, few low-income midlife women participate in weight loss maintenance trials. This pilot study aims to assess the effectiveness of two weight loss maintenance interventions in this under-represented population.

**Methods:**

Low-income midlife women who completed a 16-week weight loss intervention and lost ≥ 8 lbs (3.6 kg) were eligible to enroll in one of two 12-month maintenance programs. The programs were similar in content and had the same number of total contacts, but were different in the contact modality (*Phone + Face-to-Face* vs. *Face-to-Face Only*). Two criteria were used to assess successful weight loss maintenance at 12 months: (1) retaining a loss of ≥ 5% of body weight from the start of the weight loss phase and (2) a change in body weight of < 3%, from the start to the end of the maintenance program. Outcome measures of changes in physiologic and psychosocial factors, and evaluations of process measures and program acceptability (measured at 12 months) are also reported. For categorical variables, likelihood ratio or Fisher’s Exact (for small samples) tests were used to evaluate statistically significant relationships; for continuous variables, t-tests or their equivalents were used to assess differences between means and also to identify correlates of weight loss maintenance.

**Results:**

Overall, during the 12-month maintenance period, 41% (24/58) of participants maintained a loss of ≥ 5% of initial weight and 43% (25/58) had a <3% change in weight. None of the comparisons between the two maintenance programs were statistically significant. However, improvements in blood pressure and dietary behaviors remained significant at the end of the 12-month maintenance period for participants in both programs. Participant attendance and acceptability were high for both programs.

**Conclusions:**

The effectiveness of two pilot 12-month maintenance interventions provides support for further research in weight loss maintenance among high-risk, low-income women.

**Trial registration:**

ClinicalTrials.gov Identifier: NCT00288301

## Background

In the US, the prevalence of overweight (Body Mass Index (BMI) ≥ 25 kg/m^2^) and obesity (BMI ≥ 30 kg/m^2^) remains a serious public health problem. Middle-age (over the age of 40) and low socioeconomic status (SES) are independent risk factors for overweight and obesity in women [1,2]. Rates of obesity among women 40–59 years of age are higher than rates for younger women, with particularly high rates among Non-Hispanic Blacks and Mexican Americans [1]. When obesity rates are categorized by SES (generally measured by income and education), we observe among women a trend where less educated women are more likely to be obese compared to women with college degrees [3]. Likewise, when income and obesity rates are compared, women with incomes < 200% of poverty had higher rates of obesity than those 200% of poverty or higher [2]. Among adults, food insecurity has also been shown to be associated with obesity. Compared with food secure adults, food insecure adults had significantly higher rates of obesity if they were women ≥30 years of age, non-Hispanic whites, non-Hispanic blacks, or had a household income of < $25,000 [4]. Other measures of SES, such as residential property values also provide support for this association between SES and obesity rates in the US. In one report, women in the lowest quartile of property values were 3.4 times more likely to be obese than women in the top quartile [5]. Moreover, this inverse association between property values and obesity was independent of other SES factors [5]. All together, these data show the high risk for obesity in low-income midlife women. This high risk status does not, however, translate into greater research focus. In general, middle-aged women are well represented in the weight loss and weight loss maintenance literature [6-12], but seldom are low-income groups targeted. As a result, there is very little evidence on how to efficiently and effectively promote and maintain weight loss for this high risk population [13].

Weight-Wise (WW), a 16-week evidence-based behavioral weight loss program [6,8,9,14] was specifically designed to fill this evidence gap by targeting low-income midlife women. The WW program was tested through a randomized control trial conducted at a federally qualified community health center [15]. Despite the high effectiveness of WW (reported elsewhere [15]), questions remained about participants’ ability to maintain their weight losses, which is a constant challenge in the absence of a structured weight loss maintenance program [10,16].

To address this concern, a 12-month pilot study comparing two weight loss maintenance interventions was designed for qualifying WW participants (women who lost at least 8 lbs (3.6 kg)). The 12-month outcomes of the two pilot weight loss maintenance interventions are reported here and include evaluations of overall weight loss maintenance and several secondary outcome measures including blood pressure, dietary intake, HDL-cholesterol, as well as program attendance and acceptability.

## Methods

### Weight-wise program

Complete details concerning study design, recruitment and outcomes for the WW weight loss trial (which directly preceded the weight loss maintenance pilot) have previously been described [15]. In brief, women completing a CVD risk reduction study (that was not focused on weight-loss) [17], were invited to participate in WW during their final study visit. In addition, participants were sought through referrals from clinicians, from the greater community, and at community events. Eligibility to participate in WW included women ages 40–64 with a gross income ≤200% of the federal poverty level, uninsured or under-insured, and a body mass index (BMI) of 25–45 kg/m^2^ inclusive. Exclusion criteria included: any medical condition for which weight loss was contraindicated; CVD event in the past 3 months; and pregnancy, breastfeeding, or planning a pregnancy before the end of the study period. WW participants were randomized to one of two arms; Special Intervention (SI, n = 72) and Delayed Control (DC, n = 71). SI participants received 16 weekly group education sessions held at a local church while DC participants received two mailed newsletters focusing on non-weight related health topics. DC participants received the study intervention upon completion of the trial. A total of 143 women were recruited and enrolled in WW, and 94% (135/143) of women completed the 16-week weight-loss intervention with women in the SI group losing significantly more weight than the DC group (4.4 kg difference between groups, 95% CI (3.2-5.5), P < 0.001)[15].

### Weight loss maintenance programs

**Figure 1:**
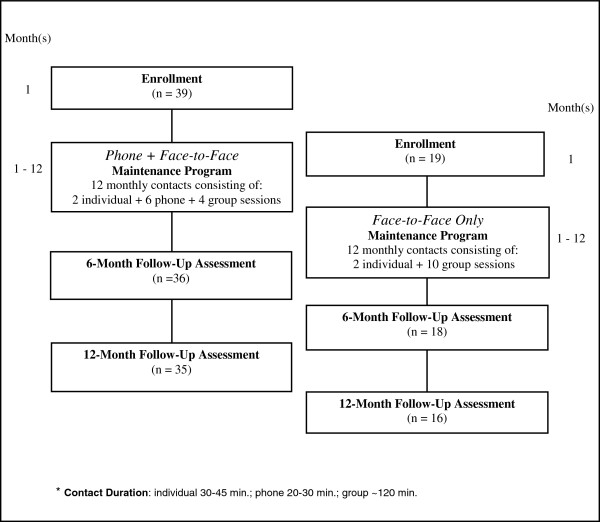
**Maintenance intervention flow diagram.** Study design for the Weight-Wise pilot maintenance programs, conducted in Wilmington, NC. Maintenance programs were implemented between September 2005 - February 2007.

To be eligible for the maintenance intervention, WW participants must have lost at least 8 lbs (3.6 kg) during the 16-week weight loss intervention phase. The 8-lb cutoff was based on the a mean weight loss rate of 0.5 lbs./ week. Weight loss rates of 0.5 to 2.0 lbs./week are generally recommended in behavioral weight loss interventions [18], and this cutoff represents the minimum expected weight loss for a 16-week program. The goal was for eligible women to start the maintenance program within 4 weeks of finishing the WW program. Hence the delayed treatment design of WW necessitated different start times for the SI and DC groups thereby prohibiting re-randomization of participants prior to beginning the weight loss maintenance phase. Figure 1 diagrams the study design of the maintenance phases for both groups. The weight loss maintenance programs are described in greater detail below.

Starting with the same basic content from WW, we developed two maintenance programs designed to be implemented using contacts primarily by group or by phone. Program content, total number of contacts and contact frequency (1 contact per month) were the same for both groups. However, the format in which the content was delivered and contact time varied. Eligible participants in the SI weight loss group (hereafter referred to as the “*Phone + Face-to-Face*” maintenance group) received a 12-month weight loss maintenance program consisting of 2 individual face-to-face contacts at data collection visits (30–45 minutes each, months 1 and 6), 4 face-to-face group contacts (120 minutes each, months 4, 8, 10 and 12) and 6 phone contacts (20–30 minutes each, months 2, 3, 5, 7, 9 and 11 ). The total intervention dose planned for the *Phone + Face-to-Face* maintenance program was 10–11 hours (excluding the time spent in data collection visits). DC participants (hereafter referred to as the “*Face-to-Face Only*” maintenance group) started their maintenance program about 5 months after the *Phone + Face-to-Face* participants, and received a 12-month program consisting of 2 individual face-to-face contacts at data collection visits (30–45 minutes each, months 1 and 6) and 10 face-to-face group contacts (120 minutes each, months 2–5 and 7–12). The *Face-to-Face Only* maintenance program included 20 hours of planned intervention contacts (excluding the time spent in data collection visits). The same health counselor from the weight loss intervention phase delivered all contacts for both maintenance programs between September 2005 and February 2007. The University of North Carolina Public Health Institutional Review Board approved and monitored the study.

During both phone and group contacts, the health counselor used a facilitator guide and covered behavioral principles important to weight loss maintenance such as relapse prevention, problem solving, stress management, enhancing motivation, and social support. The group session format consisted of a check-in with review of progress component, discussion of a behavioral topic related to weight loss maintenance, nutrition and physical activity hands-on experience with discussion, and a goal-setting with action-planning segment at the end of the session. Participant feedback reports were provided every 4 months during the maintenance program. These reports summarized weight change, weekly PA minutes, and number of food records completed weekly. Since phone contacts were shorter in duration, each component of the call (e.g., check-in, educational or behavioral content, and goal-setting with action planning) took less time and educational needs were handled mainly through brief overviews and by referring participants to program materials or other resources.

### Data collection

Prior to starting the randomized controlled trial, written informed consent and baseline measures were obtained from all participants. Measures relevant to this report on weight loss maintenance are listed below. A detailed description of all measures collected at baseline and throughout the 16-week intervention is published elsewhere [15].

#### *Physiologic measures*

Physiologic measures were collected three times during the weight loss maintenance phase (at the start, 6-months, and 12-months). Measurements included weight, blood pressure, and percent body fat (measured by bioelectrical impedance analysis (BIA)). We measured weight with electronic scales (Seca 770, Seca Corporation, Columbia, MD). Height was measured with a portable stadiometer (Schorr Productions, Olney, MD) and blood pressure (BP) with Omron HEM-907 automated BP monitor (Omron Healthcare, Inc., Vernon Hills, IL). A Tanita TBF-310 analyzer (Tanita, Arlington Heights, IL) was used to measure body composition.

#### *Weight loss maintenance definitions*

For this reporting, we defined “successful weight loss maintenance” two ways: retaining a weight loss ≥ 5% of initial weight at start of the weight loss program [19]; and a change in weight of < 3% from the start to end of the 12-month maintenance period [16]. Both definitions of ‘maintenance’ are consistent with current definitions used in weight loss studies. For the DI-Maintenance group, *initial weight* is defined as weight measured immediately before starting the weight loss program (not the weight assessed before randomization).

### Statistical methods

SAS version 9.1 (SAS Institute, Cary, NC) was used for statistical analysis. For assessing the outcome of weight loss maintenance at 12-month follow-up, we used descriptive analyses that included means and percentages, and their distributions and standard errors. For categorical variables, likelihood ratio or Fisher’s Exact (for small samples) tests were used to evaluate statistically significant relationships; for continuous variables, t-tests or their equivalents were used to assess differences between means and also to identify correlates of weight loss maintenance. All reported P values are two-sided, with significance set at 0.05.

## Results

The 58 maintenance program participants represent 43% (58/135) of participants who completed the weight loss program and lost ≥3.6 kg. Among the maintenance participants, 39 were SI participants, while 19 were DC participants. In comparing baseline demographic and physiologic characteristics of the maintenance-eligible (n = 58) vs. ineligible participants (n = 77), we found three statistically significant differences. Participants eligible for the maintenance programs were more likely to be Non-Hispanic whites (p < 0.01), not have diagnosed hypertension (p < 0.05), and have a lower percent body fat (p < 0.05). However, no significant differences were found between the *Face-to-Face Only* (DC participants) and the *Phone + Face-to-Face* (SI participants) maintenance group participants.

**Figure 2:**
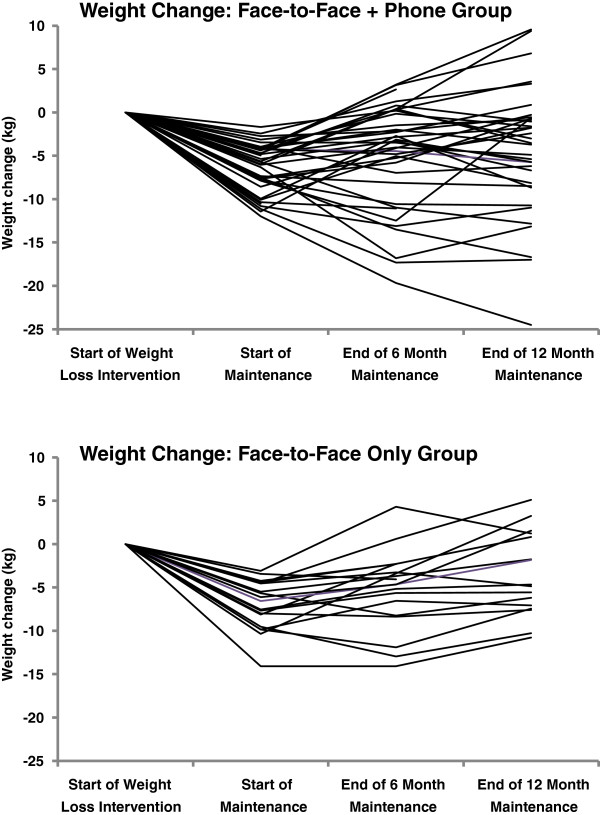
**Weight change in maintenance groups.** Weight loss from the start of weight loss to the end of the maintenance period, among participants providing weight measures at follow-up visits.

Table 1 summarizes, and Figure 2 depicts the changes in weight loss and weight regain over the 12-month maintenance period, by group. Overall, 93% (54/58) of participants provided weight measures at 6 months, and 88% (51/58) at 12 months. Participants in both the *Face-to-Face + Phone* and *Face-to-Face Only* groups began the maintenance program with similar weight loss (-6.3 vs. 7.0 kg, p = 0.37). At the end of the maintenance programs, both groups had a net weight loss of about 4 kg, with non-significant differences between groups (p = 0.65). The pattern of weight regain during the first 6 months of maintenance was also similar in both groups (p = 0.58) and averaged 1.5 kg. In contrast, the average regain during the second six-month period was much higher among *Face-to-Face Only* participants compared to *Face-to-Face + Phone* (2.0 kg vs. 0.7 kg, p = 0.24). At the end of the 12-month maintenance period the total regain among *Face-to-Face Only* participants was 4.1 kg compared to 1.7 kg in the *Face-to-Face + Phone* group (p = 0.17). Figure 2 more clearly shows this difference in weight regain. Among *Face-to-Face Only* participants, dramatic weight regains were not offset by periods of weight loss, as observed among *Face-to-Face + Phone* participants.

**Table 1:** **Weight outcomes of two 12-month weight loss maintenance programs**^**a**^

	***Face-to-Face + Phone***			***Face-to-Face Only***			
**Weight (kg)**^**b**^	**N**	**Mean**	**SD**	**N**	**Mean**	**SD**	***P-Value***
Start of weight loss intervention	39	89.0	(15.6)	19	86.7	(15.1)	0.60
Start of maintenance program	39	82.7	(14.6)	19	79.7	(14.7)	0.46
End of 6 months of maintenance	36	84.0	(14.6)	18	80.7	(16.5)	0.46
End of 12 months of maintenance	35	84.1	(15.6)	16	85.1	(17.2)	0.82
**Weight Change (kg)**^**c**^							
Start of maintenance program	39	-6.3	(2.8)	19	-7.0	(2.8)	0.37
End of 6 months of maintenance	36	-5.1	(6.0)	18	-4.7	(4.1)	0.82
End of 12 months of maintenance	35	-4.4	(7.2)	16	-3.5	(4.8)	0.65
**Weight Regain (kg)**							
End of 6 months of maintenance	36	1.2	(4.7)	18	1.9	(3.1)	0.58
End of 12 months of maintenance	35	1.7	(6.0)	16	4.1	(4.1)	0.17

^a^ Data are expressed as N, mean, and (SD) unless otherwise indicated. Weight outcomes are for participants in the Weight-Wise Program, in Wilmington, NC during the period of September 2005 to February 2007.

^b^ The weight loss and regain values will not match the differences obtained using the weights in this table due to different Ns at different measurement times.

^c^ Weight change is based on the weight loss intervention start weight.

Successful weight loss maintenance has been defined in terms of weight status 1-year post-weight loss intervention [19], with a maintained weight loss of ≥5% of initial weight as a commonly used endpoint to describe weight loss success [19]. Others define weight loss maintenance as a change in maintenance start weight of <3%, irrespective of initial weight loss [20]. The overall proportion of participants who maintained ≥5% weight loss relative to start weight was 47% (24/51) of those measured or 41% (24/58) of the maintenance-eligible group. When weight loss maintenance is defined as a weight change of < 3% (relative to the start of maintenance), 43% (25/58) maintained their weight overall, with higher rates (58% [11/19]) among *Face-to-Face Only* participants. There was considerable overlap of participants representing ‘weight maintainers’ by these two definitions. Nineteen of the 24 participants (79%) who maintained ≥5% weight loss, also had a weight change of < 3%.

As a proportion of the total study population (excluding withdrawals) at the start of the maintenance programs (n = 135), 18% succeeded in maintaining a weight loss of ≥5%. Overall rates were the same when using the standard of < 3% change in maintenance program start weight. Moreover, at the conclusion of the maintenance program, 74% (43/58) weighed less than their weight at the start of the weight loss program. When the *Face-to-Face + Phone* and *Face-to-Face Only* maintenance programs are compared by start weight, weight loss, or regain, no statistically significant differences were found between program outcomes. Likewise, there were no significant differences between groups when using either definition of weight loss maintenance.

Related to the results presented in Table 1, we also assessed factors associated with success at 12 months in keeping ≥5% of initial weight off. We looked at ethnicity, age, education, and initial weight loss during the 16-week program, BMI, and the presence of diagnosed hypertension or diabetes. Even though a larger proportion of participants who had diagnosed hypertension succeeded in maintaining a weight loss of 5% or more (48% vs. 37% among non-diagnosed; χ^2^ = 2.4, p = 0.12), this difference was not statistically significant (but likely due to the small sample size). None of the other associations were statistically significant.

While our main outcome for evaluation was weight loss maintenance, we also assessed a number of secondary outcomes and evaluated a few measures of the implementation process (attendance and program acceptability). Table 2 shows the comparison of outcomes in physiologic and psychosocial factors between *Face-to-Face + Phone* and *Face-to-Face Only* maintenance programs participants. While none of the comparisons between groups was found to be significant, some changes within groups represent statistically significant improvements. These include blood pressure and dietary changes in *Face-to-Face + Phone* and *Face-to-Face Only* participants and statistically significant improvements in HDL among *Face-to-Face + Phone* participants only. Changes in health-related quality of life (mental and physical) were not significantly different within either intervention group.

**Table 2:** Comparison of maintenance programs at 12 months (change in values from start of weight loss to end of maintenance programs)

**Variable**	***Face-to-Face + Phone (n = 35)***	***Face-to-Face Only *(n = 16)**	**P-Value**
**Physiologic Outcomes**			
Systolic Blood Pressure	-6.2 (15.3)	-11.3 (18.7)	0.31
Diastolic Blood Pressure	-5.5 (9.6)	-8.4 (11.4)	0.35
Total Cholesterol	-4.1 (36.7)	-8.9 (49.2)	0.70
HDL Cholesterol	4.7 (8.0)	3.7 (11.4)	0.72
**Lifestyle and Psychosocial Outcomes**^**a**^			
Physical Activity Assessment score (self-reported)	1.8 (7.2)	2.3 (7.7)	0.85
Dietary Risk Assessment (DRA) total score	-6.2 (9.2)	-6.0 (9.1)	0.94
Quality of Life-Physical well-being	2.4 (12.7)	-0.3 (9.9)	0.45
Quality of Life-Mental Well-Being	1.3 (10.0)	2.6 (8.1)	0.67

^a^ Change in values for participants in the Weight-Wise Program, in Wilmington, NC during the period of September 2005 to February 2007. Values are mean (SD). Negative changes in DRA scores represent improvements through lowering of dietary risk. Positive changes in physical activity, depressive symptoms, and quality of life scores represent improvements.

In our process evaluation (data not shown), we compared both maintenance programs in the number of program contacts completed and their acceptability. We found no significant differences between the programs. Overall attendance was similar in both maintenance programs. Participants in the *Face-to-Face + Phone* group received on average a total of 9.3 [SD 2.5] contacts while those in the *Face-to-Face Only* group completed an average of 9.7 [SD 2.8] of 12 total contacts. Looking at just the intervention contacts, participants in the *Face-to-Face + Phone* program completed on average 5.1 [SD 1.6] of 6 planned phone contacts and 2.7 [SD 1.1] of 4 group sessions. When asked at 12 months to rate their overall satisfaction with the maintenance program on a scale from 1 to 5 with 1 = “not very satisfied” and 5 = “very satisfied”, 91% (49/54) of respondents rated their level of satisfaction as 4 or 5. Satisfaction ratings were similar between groups.

## Discussion

In a small sample of mid-life, low-income women who completed a trial of a 16-week weight loss intervention followed by a 12-month maintenance program with monthly contacts, 18% succeeded in maintaining what would be considered clinically meaningful weight loss [21]. Our study results, though not compared to outcomes in a control group, are comparable to success rates of 20% observed in previous research of 1-year weight loss maintenance after intentional weight loss [21]. In a recent 2.5-year weight loss maintenance trial (WLM) [22], 42% of participants receiving monthly personal contacts (phone and face-to-face) maintained weight losses of at least 5% of initial weight, while 36% were within < 3% of their maintenance program start weight [22]. While we observed a similar rate of 41-43% (for both definitions of maintenance), our study duration was much shorter. Our 12-month weight regain of 2.5 kg overall is, however, similar to the approximately 2 kg regain observed in the WLM personal contact group at 12-months of maintenance [22]. Compared to the average regain of one-third of lost weight within 1-year of treatment [23], our overall weight regain of 38% is comparable, given our sample of high risk participants.

A recent review of randomized clinical trials of weight loss maintenance [10], showed effect sizes ranging from 0.01 to 0.30 for differences between treatment and control groups, in studies without medication treatment. Even though this pilot study did not include a control group, if program outcomes are compared, the observed differences in total weight regain translate into an effect size of 0.23. This pilot, like many of the weight loss maintenance trials in the review by Turk and colleagues [10], was underpowered to detect a difference in treatment effect.

With so few published weight loss maintenance studies among low-income women, it was difficult to find any comparable studies. One weight loss maintenance program for low-income minority women evaluated in a primary care setting [11] provided some data for comparison. In this study by Martin and colleagues, the difference between the treatment group and usual care (one year after a 6-month weight loss intervention) was a mean (SD) weight loss of -0.49 kg (3.3) in the treatment group and a weight gain of 0.07 (3.75) in usual care (with an effect size of 0.07). Additionally, only 7% of the maintenance intervention group had a weight loss of ≥ 5% of initial body weight at the 1-year end point [11]. This was, however, a very low-dose intervention delivered in a different setting from our pilot program, thus making it difficult to directly compare outcomes.

We pilot tested two differently formatted maintenance programs and found that they appear to be similarly effective, even though the *Face-to-Face + Phone* program had about half the intervention dose (up to 11 hours of planned contacts) compared to the *Face-to-Face Only* program (20 hours total). There was over a 2 kg difference in total weight regain between programs, suggesting that the *Face-to-Face + Phone* program may have produced better maintenance outcomes. Statistical significance was likely affected by the small sample size. With strong evidence that some type of weight loss maintenance treatment is needed to prevent weight regain after intentional weight loss [12,16], the next step is to identify cost-effective program options. Even though we did not evaluate the cost of our pilot programs, interventions delivered by phone generally cost less than face-to-face contacts (both in terms of program costs and costs to the participants [10,12]. Combining phone and face-to-face group contacts brings together the cost advantages of phone-delivery and the benefits of group interactions (and enhanced social support), which seem to be important in interventions among low-income and minority populations. Probably the best evidence of effective long-term weight loss maintenance comes from the research of Perri and colleagues [12], where weight regained during a 1-year maintenance program was 1.2 kg in both the telephone-delivered and face-to-face formats. Some of the key features of this maintenance intervention include biweekly instead of monthly contacts, use of a problem-solving approach, and emphasis on self-monitoring. Translating this evidence to fit the needs of low-income women is an important next step in weight loss maintenance research.

Beyond the weight outcomes observed in this pilot study, implementing these programs gives us important information about feasibility and acceptability. In both programs attendance was good (nearly 80% overall), and program acceptability high. More importantly, improvements in cardiovascular risk factors (e.g., blood pressure and self-reported dietary changes in both groups and HDL cholesterol in *Face-to-Face + Phone* only) remained significant (within groups) at the end of the maintenance period. These sustained improvements in blood pressure even with some relapse in weight loss maintenance, are consistent with the findings from previous trials where short-term weight loss, even if not fully maintained, was protective relative to hypertension, in the longer-term [24,25].

Even though we successfully implemented 2 weight loss maintenance interventions in a small sample of low-income midlife women, this pilot study has limitations that should be mentioned. Our pilot data are limited not only by the lack of a control group, but also by the small sample, and exclusion of men (which limits generalizability). Also, our two maintenance interventions were implemented at different time periods and may be subject to differential secular trends. Despite these limitations, this pilot study among a population group that is seldom studied, but at high risk for the negative consequences of obesity, shows promising outcomes in its high participant retention, intervention receipt and acceptability, and maintenance effectiveness.

## Conclusion

We designed and pilot-tested a weight loss maintenance program for low-income, midlife women with limited health care access. Our process data and short-term weight loss maintenance rates are very encouraging and suggest the need for further study using a randomized controlled trial study design. Further research is needed to assess longer-term weight loss maintenance outcomes and intervention designs for both weight loss and weight loss maintenance that fit the needs of low-income and healthcare underserved populations. Since the health benefits of weight loss are contingent on keeping the weight off, and low-income women have high rates of obesity with the concomitant health consequences, these results support the feasibility and need for further weight loss and weight loss maintenance research in this population. The challenge will be to conduct high-quality trials that aim to identify the optimal combination of contact formats and interval(s) between contacts, while considering relevant social context factors.

## Competing interests

Authors have no competing interests to declare.

## Authors’ contributions

CSH conceived of the study, and participated in its design and coordination, interpreted data, and drafted and revised the manuscript. LFJ participated in study coordination, data analysis and drafted portions of the manuscript. ZG conducted the statistical analysis. BAG participated in study coordination and drafted portions of the manuscript. SCL carried out the intervention and participated in data acquisition. ADG participated in study coordination and drafted portions of the manuscript. DFB drafted and revised portions of the manuscript. TCK participated in the design and coordination of the study and helped draft the manuscript. All authors read and approved of the final manuscript.

## Pre-publication history

The pre-publication history for this paper can be accessed here:

http://www.biomedcentral.com/1471-2458/13/653/prepub
